# Not all predicted CRISPR–Cas systems are equal: isolated *cas* genes and classes of CRISPR like elements

**DOI:** 10.1186/s12859-017-1512-4

**Published:** 2017-02-06

**Authors:** Quan Zhang, Yuzhen Ye

**Affiliations:** School of Informatics and Computing, Indiana University, 150 S. Woodlawn Ave, Bloomington, IN 47405 USA

**Keywords:** CRISPR–Cas system, false-CRISPR, Tandem repeat, STAR-like element

## Abstract

**Background:**

The CRISPR–Cas systems in prokaryotes are RNA-guided immune systems that target and deactivate foreign nucleic acids. A typical CRISPR–Cas system consists of a CRISPR array of repeat and spacer units, and a locus of *cas* genes. The CRISPR and the *cas* locus are often located next to each other in the genomes. However, there is no quantitative estimate of the co-location. In addition, ad-hoc studies have shown that some non-CRISPR genomic elements contain repeat-spacer-like structures and are mistaken as CRISPRs.

**Results:**

Using available genome sequences, we observed that a significant number of genomes have isolated *cas* loci and/or CRISPRs. We found that 11%, 22% and 28% of the type I, II and III *cas* loci are isolated (without CRISPRs in the same genomes at all or with CRISPRs distant in the genomes), respectively. We identified a large number of genomic elements that superficially reassemble CRISPRs but don’t contain diverse spacers and have no companion *cas* genes. We called these elements false-CRISPRs and further classified them into groups, including tandem repeats and *Staphylococcus aureus repeat* (STAR)-like elements.

**Conclusion:**

This is the first systematic study to collect and characterize false-CRISPR elements. We demonstrated that false-CRISPRs could be used to reduce the false annotation of CRISPRs, therefore showing them to be useful for improving the annotation of CRISPR–Cas systems.

**Electronic supplementary material:**

The online version of this article (doi:10.1186/s12859-017-1512-4) contains supplementary material, which is available to authorized users.

## Background

Phages are believed to largely outnumber their bacterial hosts in the ecosystems [1, 2] and thus pose a significant impact on the diversification of bacteria. On the other hand, bacteria develop various defense mechanisms, such as innate and adaptive immunities to protect them against invading nucleic acids including phages and other elements such as plasmids and genomic islands. The CRISPR–Cas (clustered, regularly interspaced short palindromic repeats–CRISPR-associated proteins) adaptive immune system is one of the mechanisms that prokaryotes have evolved to defend against invaders. The CRISPR–Cas systems are widespread in prokaryote, and have been found in most of the archaea species and about half of the bacterial species [3–5].

The typical genomic architecture of a CRISPR–Cas locus is composed of a CRISPR array, a locus of *cas* genes, and a leader region. Generally in a CRISPR array, the nearly identical repeats (the length of a repeat is from 21 to 47 bps) are separated by spacers of similar sizes: the spacers are the unique fragments acquired from foreign nucleic acid sequences. The leader sequence is an AT rich ~100-500 bp nucleotide sequence, and it is believed to serve as a promoter element for its adjacent CRISPR transcription [6] (and internal promoters are found within some CRISPRs [7, 8]). The defense activity of the CRISPR-Cas systems involves three steps: the acquisition of new spacers (the adaptation stage), biogenesis of crRNAs (the CRISPR transcripts), and the interference against cognate invaders guided by crRNAs [9]. During the adaptation stage, the targeted nucleic acid sequence from the invader is integrated into the CRISPR array with the help of Cas proteins, such as Cas1, Cas2 as nuclease proteins [10]. During the expression and interference stages, the precursor CRISPR locus (pre-crRNA) is then transcribed and processed into short mature CRISPR RNAs (crRNAs). Together with a Cas protein complex or a single Cas protein—depending on the different type of interference mechanism (see below)—the crRNA is guided to detect and further degrade the target DNA or RNA that contains the complementary sequence of the spacer [4, 11–13].

At the broadest level, the CRISPR-Cas systems can be divided into two classes. The class 1 system performs the function by a multisubunit Cas protein complex, and the class 2 system requires only a single Cas protein (Cas9 or Cpf1) in the crRNA-effector complex [14]. The class 1 includes type I, III, and IV systems, and the class 2 includes type II and V systems [14]. The signature genes of type I-V systems are *cas3*, *cas9*, *cas10*, *csf1*, and *cpf1*, respectively. Five main types can be further divided into 16 distinct subtypes: types I A–F and U, types II A–C, types III A–D, a type IV and a type V based on the different combination of additional *cas* genes [4, 14, 15]. Type I and II CRISPR-Cas systems provide the immunity against DNA [16, 17], whereas type III CRISPR-Cas systems are believed to target either DNA or RNA (e.g., *Streptococcus thermophiles* DGCC8004 Csm (III-A) complex (StCsm) has been demonstrated targets RNA [18]). The Cpf1-family protein found in type V (class 2) CRISPR-Cas systems has been experimentally demonstrated to perform DNA interference in a recent study [19].

The *cas* genes are usually believed to present in the direct vicinity of CRISPR loci [20]; and in the cases when multiple CRISPR arrays exist, some may be distant to the *cas* genes. Isolated CRISPRs, which lack nearby *cas* genes, were identified in a few species including *Listeria monocytogenes* [21], *Aggregatibacter actinomycetemcomitans* [22], and *Enterococcus faecalis* [23]. Some of these isolated CRISPRs were observed to be expressed but not processed into small crRNA (e.g., in *L. monocytogenes*), which indicates they may be the remnants of previous functional CRISPR–Cas systems [14] or be involved in the bacterial autoimmunity [21]. The spacer sequences in the orphan CRISPRs found in *A. actinomycetemcomitans* were antisense to bacterial self-coding genes [22], which further suggests that the existence of orphan CRISPRs is related to the regulation of other gene expression [24]. In *Haloferax volcanii*, which contains three CRISPR loci with almost identical repeat sequences, all three CRISPR loci were expressed, producing CRISPR RNA (crRNA); however, it was found that not all crRNAs can trigger successful interference [25].

Here we systematically examined the genomic location of the CRISPR–Cas systems in the bacterial complete and draft genomes to quantify the tendency of co-localization of CRISPR array and *cas* genes, taking advantage of the recently updated classification of Cas proteins by Koonin and colleagues [14]. We further explored the possible explanations to the existence of isolated *cas* loci using representative species. From isolated CRISPRs (without companion *cas* genes), we collected highly suspicious CRISPRs that lack any spacer diversity (and therefore unlikely to be real CRISPRs) and named them false-CRISPR elements. It has been shown that some tandem repeats may be confused as CRISPRs as some of them may contain “repeat-spacer” like structures [26], and *Staphylococcus aureus* repeat (STAR-like) elements (GC-rich direct repeats) could be confused as CRISPRs in *Staphylococcus aureus* [27, 28]. No study, however, has been carried out to systematically characterize these false-CRISPRs. We therefore classified the false-CRISPRs we identified into three categories based on their distribution in the genomes and “spacer” diversity: tandem repeats, STAR-like elements, and simple repeats. We note that some false-CRISPR elements were reported as CRISPRs in previous studies [29–32]. We believe this would pose a severe problem if they get propagated into downstream analysis and annotations.

## Methods

### Identifying CRISPR-Cas systems in bacterial genomes

We first used MetaCRT [33], which we modified from CRT [34] (to allow detection of partial repeats at the ends of CRISPR arrays), to predict the CRISPR arrays in complete bacterial and archaeal genomes. The genomes were downloaded in October 2016 from the NCBI ftp website (ftp://ftp.ncbi.nlm.nih.gov/genomes/refseq). We focused on complete reference genomes in this study, as CRISPR–Cas systems may be found in separate contigs when draft genomes are used. However, for a few species we analyzed in detail, we augmented the list of genomes with draft genomes: including 13 draft genomes for *Streptococcus thermophilus* and 4055 draft genomes for *Staphylococcus aureus.* In some cases, a long CRISPR may be split into multiple ones because of repeats containing excessive mutations or long spacers. To avoid such cases, CRISPRs that are close to each other (<=200 bps) and share very similar repeat sequences were considered to be in the same locus. We then collected the consensus repeat for each putative CRISPR array. We clustered these consensus repeats at 90% sequence identity using CD-HIT-EST [35]. In this way, a “cluster” contains more than two CRISPR arrays, and a “singleton” refers to the repeats exclusively found within their corresponding CRISPR array.

We then used hmmscan [36] to search putative proteins found in the genomes against a collection of Cas families to predict putative Cas proteins (using the gathering cutoff). In total, the collection contains 403 Cas families, among which eight were identified from the human microbiomes (using a combination of context-based and similarity-search approaches) [37], and 395 were from a recent study [14]. Since Koonin and colleagues did not build models for the Cas families they curated [14], we used hmmbuild to construct hmm models for all of their families. Considering that gene prediction is far from perfect for many genomes, for the genomes/contigs that contain CRISPRs but lack *cas* genes, we further used the FragGeneScan [38], a gene predictor we have developed for predicting complete as well as fragmented genes in genomic sequences, to re-predict the genes, and then performed *cas* gene prediction to rule out the possibility of missing *cas* genes because the genes were not predicted in the first place.

A *cas* locus defined in this study should contain at least three *cas* genes, at least one of which belongs to the universal *cas* genes for CRISPR adaptation (*cas1* and *cas2*) or the main components of interference module including *cas7*, *cas5*, *cas8*, *cas10*, *csf1*, *cas9*, *cpf1* [14].

### Determining the type of CRISPR-Cas loci

The CRISPR(s), together with its nearby (within 10,000 bps) *cas* genes, are defined as a CRISPR-Cas locus. A CRISPR that lacks *cas* genes in its vicinity region is defined as an isolated CRISPR locus. Conversely, a *cas* locus that does not have a nearby CRISPR array is called an isolated *cas* locus. The type of each CRISPR-Cas locus is determined according to type signature *cas* genes [4]. We say the type assignment of a *cas* locus is confident if it has at least three type-consistent signature *cas* genes, except for type V. Since only one signature gene *cpf1* is reported for type V [14], we assign type V based on a single signature gene, *cpf1*.

### Calculating spacer diversity of a CRISPR

Spacers in a true CRISPR array are likely to be distinct (e.g., only two redundant spacers were found among the total 70 spacers in the long CRISPR array in the *Streptococcus mutans* NN2025 genome). Spacer diversity, therefore, has been used as one of the indications of the activity of CRISPR–Cas systems [39]. We define that a CRISPR contains diverse spacers if at least half of its spacers share no more than 70% sequence identity by CD-HIT-EST clustering [40].

### Phylogenetic tree reconstruction

We build phylogenetic trees for selected species, using concatenated sequences of 35 marker genes predicted from their genomes [41]. To construct the phylogenetic tree, we utilized MUSCLE [42] to align the protein sequences, and applied the FastTree program [43] to construct the neighbor-joining trees using the discrete gamma model with 20 rate categories.

### Availability of our results and software

We have made our results, including the CRISPRs, false-CRISPRs (and their annotations) at the CRISPRone website (http://omics.informatics.indiana.edu/CRISPRone) for users to download. The CRISPRone website also provides online prediction of CRISPR–Cas systems given genomic sequences, using a pipeline with integrated checking of false-CRISPRs.

## Results

### Distribution of CRISPR-Cas systems in bacterial genomes

A total of 3323 and 370 *cas* loci (see in MATERIALS AND METHODS) (with or without CRISPRs in the neighborhood) were identified from 5596 bacterial and 214 archaeal complete genomes, respectively. Overall, Seventy-nine percent (2926 out of 3693) of them were confidently assigned to five main types (I-V), which includes 2001 (~68%) type I *cas* loci, 477 (~16%) type II *cas* loci (no type II *cas* loci were found in archaeal genomes, as discussed in [4]), 389 (~13%) type III *cas* loci, 24 type IV *cas* loci (no type IV *cas* loci were found in archaeal genomes), and 35 type V *cas* loci. These results suggest that the type I CRISPR-Cas system is the major type found in the bacterial genomes, which is consistent with the results in previous studies [14]. Since type IV and V CRISPR–Cas systems are rare, in the following analyses, we focused on type I, II and III systems.

It has been found that many organisms lack *cas1* and *cas2* genes in their type III CRISPR-Cas loci, but the functionality of *cas1* and *cas2* could be provided in trans from an additional *cas* locus (of either type I or type II) [4, 44]. In our study, this scenario was also observed in type I CRISPR-Cas loci (Table 1). We found 13% (263 out of 2001) of type I and 49% (191 out of 389) of type III *cas* loci are devoid of *cas1* and *cas2* genes (but not in type II systems). Among the *cas* loci lacking *cas1* and *cas2* genes, 36 type I (out of 263) and 66 (out of 389) type III *cas* loci have adjacent CRISPRs and remote *cas1* and/or *cas2* genes in the same genome, suggesting that the *cas1* and *cas2* genes may function in trans. We found 51 type I and 27 type III CRISPR-Cas loci (containing CRISPRs and other *cas* genes) lacking *cas1* and *cas2* in the genomes (but other *cas* genes still exist), which may result in losing the novel spacer acquisition ability of a CRISPR-Cas system (no alternative way has been discovered) while the interference ability may retain.

**Table 1:** Distribution of *cas1-cas2* genes pair together with CRISPR in three CRISPR-Cas system types

CRISPR	Nearby *cas*1-*cas*2	Remote *cas*1-*cas*2^a^	Type I	Type II	Type III
+	+		1651	368	187
	-	+	36	0	66
	-	-	51	0	27
-^b^	+		87	109	11
	-	+	52	0	84
	-	-	124	0	14

^a^For each CRISPR, we only checked for the presence of remote *cas*1-*cas*2 gene pair when no *cas*1-*cas*2 gene pair is found in the neighborhood of the CRISPR

^b^When lacking the CRISPRs, we examined the *cas* locus containing a nearby or remote *cas*1-*cas*2 gene pair. In this table, "+" indicates presence, "-" indicates absence, and a blank cell means the corresponding aspect was not checked

**Fig. 1:**
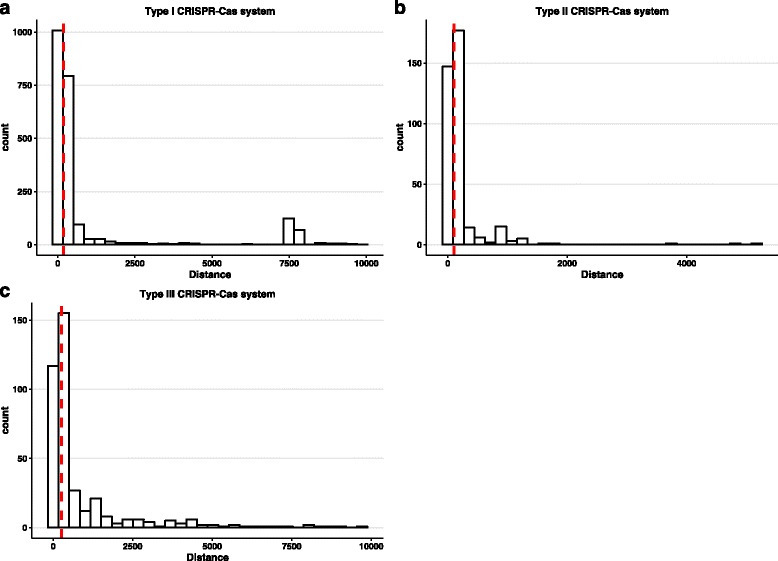
The distribution of the distance between CRISPR and the nearest *cas* gene for **a** type I, **b** type II and **c** type III systems. The *red dash line* shows the median distance

A previous study [14] has reported the distribution of the genomic distances between CRISPR arrays and *cas* loci (from four to 4,477,432 bps). However, the distance distributions for each main type have not been estimated separately. We calculated and compared the distances between a CRISPR array and its nearest *cas* gene for the three main types (Fig. 1). Note that in this analysis we only include CRISPRs and *cas* loci within a 10,000 bp window. The median distances between CRISPRs and the nearest *cas* genes are 179 bps, 103 bps, and 268 bps for type I, II and III systems, respectively. The pair-wise comparisons (Mann–Whitney u test: type I vs. type II: p-value < 2.2e-16; type I vs. type III: p-value = 3.85e-07; type II vs. type III: p-value < 2.2e-16) indicate the distributions of three types of CRISPR-Cas systems are significantly different. The results suggest that for type II systems, their CRISPRs tend to be located closer to associated *cas* locus (with shorter leader sequences) than type I and type III systems. In addition, among 24 type IV and 35 V CRISPR-Cas loci, the median distance between type IV cas locus and its CRISPR is 137 bp and 147 bps, respectively.

### Prevalence of isolated/orphan *cas* loci in bacterial genomes

Although *cas* loci and CRISPRs tend to be clustered in the same genomic neighborhood, isolated *cas* loci (or CRISPRs) are found in genomes. In this study, if a *cas* locus (containing at least three *cas* genes) has no companion CRISPR array within a 10,000 bp window, we call it an isolated locus. An isolated *cas* locus is considered an orphan if its companion CRISPR is lost from the genome. A total of 2739 (including 2555 bacterial and 184 archaeal) species each were found to contain at least one isolated *cas* locus, resulting in a total of 753 and 101 isolated *cas* loci in bacterial and archaeal gnomes, respectively. 86% (650 out of 753) of bacterial species and 31% (57 out of 184) of archaeal species harbor only one isolated *cas* locus, although some may contain as many as four of such loci. In summary, among predicted *cas* loci, 12% (236/2001) of type I, 22% (109/477) of type II, and 28% (109/389) of type III *cas* loci are found to be isolated. Type III CRISPR–Cas systems have the highest ratio of isolated *cas* loci.

Isolated *cas* loci are either remnants of CRISPR–Cas systems without the immunity function, or they function together with remote CRISPR(s) in the same genome. On the other hand, an orphan *cas* locus may be non-functional, or lose its immunity function but maintain other function(s) (it was shown that some components of the CRISPR–Cas systems have a function in DNA repair [45]). Similarly, isolated CRISPRs can be non-functional (orphan), or work with distant *cas* locus in the same genome. Below we present selected examples belonging to the different scenarios.

**Fig. 2:**
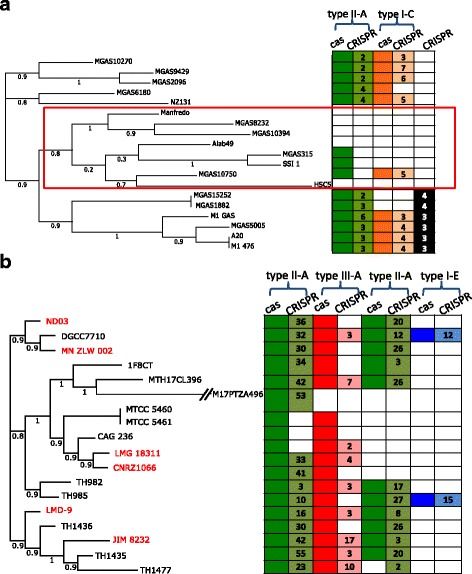
CRISPR–Cas systems in representative species: *Streptococcus pyogenes* (**a**) and *Streptococcus thermophilus* (**b**). The phylogenetic trees of the isolates are shown on the left (only strains with complete genomes are included in **a**; and in **b**, stains with complete genomes are highlighted in *red*). The tables on the right show the presence and/or absence of the individual components: colors indicate the presence, whereas *white boxes* indicate the absence. The numbers in the CRISPR columns indicate the number of spacers within corresponding CRISPR

Analysis of 49 *Streptococcus pyogenes* isolates revealed a complete type I, a complete or partial (with *cas* locus only) type II CRISPR-Cas system, and an isolated CRISPR associated with this species (Fig. 2a). 12 isolates harbor all elements, and others have some of the elements. The isolated CRISPR is likely to be an orphan that has lost its function, because 1) its repeat sequence is different from the repeats found in the type I and type II systems, and 2) no spacer turnover was observed in this isolated CRISPR—the same set of spacers are found in this CRISPR across all six isolates harboring it (except strain MGAS15252 and strain MGAS1882 each have one spacer duplication). By contrast, CRISPRs associated with type I and type II systems have diverse spacers across the different isolates. A branch (highlighted with a box in Fig. 2a) contains strains that have complete or partial loss of the type I and type II CRISPR–Cas systems: Manfredo, MGAS8232, MGAS103;94 and Alab49 have none of the systems; MGAS6180 has an incomplete type I system with *cas* locus but no CRISPR; and MGAS10750 has an incomplete type II system with *cas* locus only. Overall, the pattern of CRISPR gain and loss is consistent with the phylogenetic tree for this species (see Additional file 1 for the tree with all 49 strains), which may provide a snapshot of highly dynamic gain and loss of CRISPR-Cas systems during the evolution of the *S. pyogenes*.

The second example involves 18 *Streptococcus thermophilus* strains. The total of four CRISPR-Cas loci—including two type II-A loci with different consensus repeats (on the different strands), a type III-A system, and a type I-E system—were found in *S. thermophilus* (in Fig. 2b): the activity of two type II-A CRISPR-Cas loci was demonstrated in the previous studies [12, 39, 46, 47], and type III-A CRISPR-Cas locus has been experimentally demonstrated to target the RNA [18]. Diverse spacers are found in the CRISPRs among these 18 isolates, consistent with a previous study [39]. Complete and partial loss (resulting in isolated *cas* locus or CRISPR) of the different CRISPR–Cas systems were observed in this species—eight of the “complete” (based on Makarova et al’s definition [14]) type III-A cas loci lost their companion CRISPRs; by contrast, only three out of 29 type II-A *cas* loci do not have companion CRISPRs. This is consistent with the statistics based on the CRISPR–Cas systems in all species (see above), which showed that type III *cas* loci have the least tendency of co-locating with their companion CRISPRs among the three types of CRISPR–Cas systems.

In the last example, isolated *cas* loci found in *Zymomonas mobilis* are likely to function with remote CRISPR(s) in the same genome. Seven closely related strains (including ATCC 29191, ZM4, NCIMB 11163, ATCC 10988, 2 strains of NRRL_B-12526 and CP4 = NRRL B-14023) each harbor a *cas* locus containing type I-F signature genes, with CRISPRs distant in the genome. One strain (ATCC 29192), which is phylogenetically more distant from other strains, contains a type I-E *cas* locus and a CRISPR in the distance (Additional file 2). All CRISPRs loci of type I-F, scattered in the genomes, share the same repeat sequence. The large variety of CRISPR length and spacer sequences, together with the “complete” subtype I *cas* loci, implies that the type I *cas* loci together with the remotely CRISPR loci may still be active.

### Curation of false-CRISPRs

A total of 11,729 putative CRISPRs were predicted including 10,754 from complete bacterial and 975 from archaeal genomes. All CRISPRs are first grouped based on their consensus repeat sequences (by CD-HIT-EST using 90% as the sequence identity cutoff), resulting in a total of 1222 groups, each containing at least two CRISPRs and 2996 singletons (see Methods). Groups of putative CRISPRs are then evaluated using two criteria. (1) Are CRISPRs in a group tend to be located near *cas* genes? If not, are there *cas* loci in the same genomes though they are far from the CRISPRs? (2) Do CRISPRs contain diverse spacers?

We consider a group of putative CRISPRs containing at least one CRISPR with companion *cas* genes a group of “real” CRISPRs (their sequences are provided in Additional file 3). Therefore, all of the putative CRISPRs belonging to this group are considered to be real CRISPRs (Table 2). For example, the CRISPR found in *Aggregatibacter actinomycetemcomitans* strain 624 does not have nearby *cas* genes, but it shares similar repeat sequences (>90% sequence identity) with other CRISPRs found together with subtype I-F *cas* genes in genomes including *Actinobacillus equuli* subsp. equuli strain 19392 and *Candidatus Symbiobacter* mobilis CR. In this way, we collected 616 real CRISPR clusters (covering a total of 5676 CRISPRs). Reassuringly, almost all of these (5662/5676, 99%) real CRISPRs are found to have diverse spacers (see Table 2).

**Table 2:** Characterization of the “CRISPR” clusters according to the *cas* genes and spacer diversity

% co-location	# of clusters	# of CRISPRs						
		*cas*-near		*cas*-far		*cas genes* not found in the genome		
		d+	d-	d+	d-	d+	d-	short
Singletons	2996	477	4	767	473	689	518	68
[0,0.1)	615	4	7	1365	587	947	614	87
[0.1,0.2)	13	34	0	216	0	11	0	0
[0.2,0.3)	24	79	3	194	0	38	0	0
[0.3,0.4)	32	85	0	142	0	13	0	0
[0.4,0.5)	19	240	0	212	0	52	0	0
[0.5,0.6)	81	202	0	145	0	34	0	0
[0.6,0.7)	37	177	0	75	0	20	0	0
[0.7,0.8)	29	884	0	205	0	66	0	0
[0.8,0.9)	21	286	0	43	0	5	0	0
[0.9,1)	11	353	0	11	0	5	0	0
1	340	1292	0	0	0	0	0	0

Descriptions of the columns: “% co-location” shows the percentage of CRISPRs co-locating with *cas* genes in each cluster; “d+” represents that CRISPR contains diverse spacers, whereas “d-” indicates no spacer diversity was observed; “short” represents short CRISPRs (with two spacers without spacer diversity)

Groups of putative CRISPRs that lack evidence (i.e., without *cas* genes in the host genomes and/or spacer diversity) and are not similar to real CRISPRs (containing at least 5 mismatches compared to real CRISPR repeats), on the other hand, are likely to be the genomic elements that superficially reassemble the CRISPR’s repeat-spacer structure but are not real CRISPRs. As a result, we derived a total of 3224 such elements, called false-CRISPR elements (their consensus “repeat” sequences are shown in Additional file 4), from 366 clusters and 1723 singletons of putative “CRISPRs”.

### Annotation of false-CRISPR elements

**Fig. 3:**
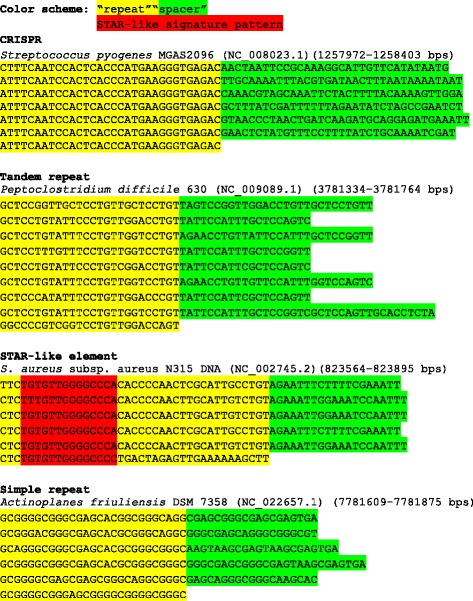
An illustration of a typical CRISPR and other genomic elements that superficially reassemble the CRISPR’s repeat-spacer structure

For each group of false-CRISPRs, we checked the spacer diversity of the “CRISPRs” in each group. Further, we applied Tandem Repeat Finder [48] and RepeatMask to check if a “CRISPR” is likely to be a tandem repeat or simple repeat due to the low complexity of DNAs. We classified false-CRISPRs into four categories: (1) tandem repeats, (2) STAR-like elements, (3) simple repeats, and (4) unknown, for the CRISPRs that don’t fall into the other three categories (false-CRISPRs and their annotations are provided in Additional file 5). See Fig. 3 for examples of the different categories, highlighting the differences of the different elements.

#### Tandem repeats

Tandem repeats are the special sequences that are abundant in prokaryotic genomes. The region containing the tandem repeats is potentially hypermutable, which allows the bacteria to adapt to changing environments without increasing overall mutation rate [49, 50]. The hypermutable tandem repeats may have very similar structure with CRISPR arrays. In total 1744 out of 3224 (54%) false-CRISPRs (from 219 clusters and 822 singletons) were predicted to be tandem repeats by Tandem Repeat Finder [48].

#### STAR-like elements

In the previous study, Cramton et al. [27] identified the *Staphylococcus aureus* repeat (STAR-like) element, which contains the extraordinarily CG-rich repeats, and this repetitive element was found in up to 21 copies in a *S. aureus* genome. The structure of STAR-like elements could easily be confused with real CRISPRs. STAR-like elements contain the signature sequence T[G/A/T]TGTTG[G/T]GGCCC[C/A] [27], We checked for this signature sequence in our collection of false-CRISPRs and found 139 of them contain this signature which were therefore classified as STAR-like elements.

#### Simple repeats

We observed that some of the false-CRISPRs contain short (1 bps - 5 bps) low-complexity repeats. Using RepeatMasker (http://www.repeatmasker.org/cgi-bin/WEBRepeatMasker), 56 false-CRISPRs were identified to contain the simple sets of DNA repeats. For example, the false CRIPSR found in *Burkholderia pseudomallei* 668 (genome ID: NC_009074; position 924,901 bps - 925,214 bps) contains 12 copies of sequence pattern GCCGTT. Six false-CRISPRs contain low complexity sequences, for example, the false-CRISPR in *S. aureus* TCH60 (genome ID: NC_017342; position 1,242,548 bps −1,242,837 bps), which is not STAR-like and tandem repeat, is identified as A-rich (43% of the region is adenine) and low complexity region.

### Real and false CRISPRs in *S. aureus*

In total, 219 CRISPRs (in 23 clusters and 17 singletons) were identified by metaCRT from 123 *S. aureus* complete genomes (i.e., all these elements have the repeat-spacer structures). Six CRISPRs (from 3 clusters) are identified as real CRISPRs in our study. The 213 others are “false” CRISPR elements, among which 53 are tandem repeats, and 136 arrays are identified as STAR-like elements. In addition, we identified 26 real CRISPRs from *S. aureus* draft genomes, which far outnumbered the complete *S. aureus* genomes.

**Fig. 4:**
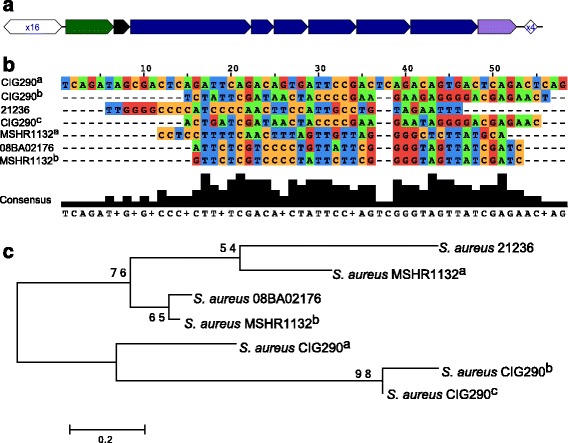
Comparison of the CRISPRs found in *S. aureus*. **a** The complete subtype III-A CRISPR-Cas systems identified in *S. aureus* 08BA02176. **b** The multiple alignments of all real CRISPRs grouped in seven clusters, using one representative repeat sequence for each cluster. *S. aureus* strain names are shown on the left. **c** The phylogenetic tree of the CRISPRs, built from the multiple alignment shown in (**b**). CIG290a represents the repeat sequence in the orphan CRISPR in *S. aureus* CIG290. CIG290b and CIG290c represent the repeat sequence in the CRISPRs that are in the downstream and upstream of the *cas* locus in *S. aureus* CIG290 (contig: NZ_AIES01000010), respectively. MSHR1132a represents the repeat sequence in the orphan CRISPR in *S. aureus* MSHR1132, whereas MSHR1132b represents the repeat sequence in a CRISPR that is in the upstream of subtype III-A *cas* locus (the distance between the MSHR1132b and the closest *cas* gene is 74 bps)

Complete subtype III-A CRISPR-Cas systems were identified in three complete genomes (08BA02176, MSHR1132, as reported in the previous study [51], and JS395*)* and two draft (CIG290 and 21252) genomes. CRISPRs are both found upstream and downstream of the *cas* locus in the same genome (see Fig. 4a for *S. aureus* 08BA02176). Other isolates share similar organization of the CRISPR–Cas systems (with two CRISPRs sandwiching a *cas* locus), but the length of the CRISPRs varies. The upstream CRISPRs contain between four (CIG290) and 16 (08BA02176) repeats, and the downstream CRISPRs contain either four or five repeats. The two CRISPRs sandwiching the *cas* locus in *S. aureus* CIG290 (contig NZ_AIES01000010) share similar repeats but with similarity less than 90%, so they were grouped into two clusters (see Fig. 4b for the alignment of the repeat sequences and Fig. 4c for the tree of the repeats built from the alignment). In addition to the two CRISPRs co-located with the *cas* locus, an orphan CRISPR was found in *S. aureus* CIG290 which also shares similar repeat with the other two CRISPRs in this genome. We note that CRISPRs found in some isolates, including *S. aureus* 21236 and *S. aurues* MSHR 1132, share more similar repeats with *S. epidermidis* than *S. aureus* CIG290.

**Fig. 5:**
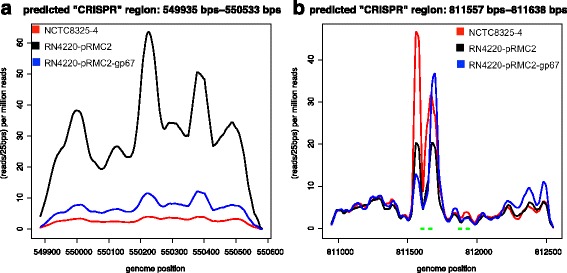
Expression of false-CRISPRs found in *S. aureus*. The expression level of the elements was measured by reads per 25 bp per million total reads and the x-axis shows the position along the *S. aureus* 8325 genome in NCTC8325-4 (*red line*), RN4220-pRMC2 (*black line*) and RN4220-pRMC2-gp67 (*blue line*) cells. **a** The short CRISPR-like element, which was reported as a “CRISPR” in [32]. **b** The CRISPR-like element having overlap with a protein-coding gene is predicted to be tandem repeats. The regions containing STAR-like elements are represented by *green lines*. To evaluate the expression level of false-CRISPRs, we used TopHat2 [55] with default parameters to align the single-end reads, which were downloaded from NCBI SRA (http://www.ncbi.nlm.nih.gov/sra/; the accession number is SRP027410), to the *S. aureus* NCTC8325 genome

Notably, one of the false-CRISPRs we identified in *S. aureus* NCTC8325 was considered as a genuine CRISPR in a previous study [32] which used high throughput RNA-sequencing (RNA-seq) to examine gene expression, including their predicted orphan “CRISPR”. In this *S. aureus* strain, we identified four false-CRISPRs including three STAR-like elements and one tandem repeat. One STAR-like element (located between 811,557 bps −811,638 bps) was mistaken as a CRISPR in Osmundson et al. [32] (shown in Fig. 5 in their paper). RNA-seq reads were found covering all three STAR-like elements, including the one studied by Osmundson et al. [32] (shown in Fig. 5a), suggesting that these elements were expressed. The tandem repeat is located between 547,751–550,738 bps within a protein-coding gene between 547,751–550,738 bps, which encodes for a fibrinogen-binding protein SdrC. This tandem repeat is found to be expressed (as shown in Fig. 5b), which is not surprising. However, the biological meaning of the other three false-CRISPRs (the STAR elements) remains to be investigated.

### False-CRISPR elements in existing collections of CRISPRs

Since most existing methods for CRISPR identification are based on finding regions with repeat-and-spacer like structures, we expect to find false-CRISPRs in the collections of CRISPRs identified using these methods. We checked for presence of false-CRISPRs in Biswas’ collection [29], CRISPRBank [30], CRISPRmap [31], and the NCBI annotations [52]. Because CRISPRmap only provides repeat sequences (but not genome and coordinate information of the repeats), we used similarity search to find false-CRISPRs in this collection: a repeat in CRISPRmap that shares 90% sequence identity, covering 90% of its length, with a false-CRISPR we identified is considered a potential false-CRISPR.

We found that 162 false-CRISPRs were collected in the early study conducted by Biswas et al [29] as CRISPRs, counting for 4.5% (out of total 3571 CRISPRs predicted in [29]) of their collection of predicted CRISPRs. Among the 162 false-CRISPRs, 68 belong to tandem repeats, and 14 are STAR-like elements (Table 3). We noticed that 104 out of the 162 (64%) false-CRISPRs had only weak evidence of transcriptional direction prediction (see Additional file 6), an indirect evidence suggesting that they are unlikely to be real CRISPRs. We checked a more recent collection of CRISPRs from Biswas et al [30]. Among 19,415 CRISPRs (each has at least two repeats of 23 bps or longer) collected in CRISPRBank (http://bioanalysis.otago.ac.nz/CRISPRBank/), 191 (~1%; out of 19,415) are similar to false CRIPSRs, and most of them (81%; 155 out of 191) were considered as weak predictions (with scores below 4.0) by CRISPRDetect [30]. Among 191 false-CRISPRs, 46 are identified as tandem repeats and 18 are classified as STAR-like elements (see Table 3).

**Table 3:** Breakdown of the false-CRISPRs found in existing collections of CRISPRs

	Biswas’ collection [29]			CRISPRBank [30]			CRISPRMap [31]		
	Total # of CRISPRs	# of clusters	# of singletons	Total # of CRISPRs	# of clusters	# of singletons	Total # of CRISPRs	# of clusters	# of singletons
Tandem repeats	68	20	39	46	22	21	21	11	6
STAR-like elements	14	2	0	18	4	0	12	4	0
Simple repeats	2	0	1	4	1	3	7	1	5
Unknown	78	17	49	123	30	77	58	14	28
Total	162	39	89	191	57	101	98	30	39

For the CRISPRmap [31] collection, 98 (out of 3527, 2.8%) repeats are similar to false-CRISPRs, among which 21 and 12 are classified as tandem repeats and STAR-like elements, respectively (Table 3). We further checked the CRISPR annotations provided by the NCBI [52] which combined CRT [30] and PILER-CR [53] to predict CIRPSRs, in archaeal and bacterial genomes. Out of 6386 CRISPR arrays (1557 from archaeal and 4829 from bacterial genomes) that were annotated in NCBI annotation files, 71 (1%; out of 6386) could be identified as false-CRISPRs.

## Discussion

In this study, we provide an overview of the distribution of different types (I-V) of CRISPR-Cas systems and also evaluate the CRISPRs and *cas* loci co-location tendency among currently available archaeal and bacterial complete genomes. Our analysis has shown that isolated CRISPRs and *cas* loci could be the remnant of the non-functional CRISPR-Cas systems, or they could function remotely with each other.

The existing, widely used CRISPR detection tools, such as CRISPRFinder [26] and CRT [34], predict the CRISPRs primarily based on the typical structure of CRISPRs (the almost identical repeats are separated by spacers). However, this structure is easily confused with other kinds of elements such as tandem repeats, STAR-like elements and simple repeats. Combing genomic context analysis and the diversity analysis of the “spacers,” we collected 3224 (~27%, 3224 out of 11,729 predicted “CRISPRs”) suspicious orphan CRISPRs, named false-CRISPRs.

Although earlier simpler prediction methods [26, 34] will predict false positives, later methods (e.g., the NCBI annotation in RefSeq [52] and CRISPRDetect [30]) have lower levels of false positives (for example, CRISPRDetect [30] has 0.2% false positives). Our results indicate that predictions of CRISPR solely based on the repeat-spacer structural patterns will pose a high risk of false positives, thus the use of additional information (*i.e.*, spacer dis-similarity), proposed both in our study and recently developed approaches including CRISPRDetect [30], could greatly improve real CRISPR identification. Since about 50% of our false-CRISPR elements are identified as tandem repeats, we believe it is a useful step to run Tandem Repeat Finder [48] to filter out CRISPR predictions. Our collection of false-CRISPR and their classifications can be utilized in further studies to reduce the false annotation of CRISPR.

There are still a significant number of false-CRISPRs (1285) that remain unknown. We found that some repeat sequences of these unknown false-CRISPRs are extremely prevalent in their corresponding genomes, which may be caused by nucleotide composition bias. For example, false-CRISPRs found in the *Conexibacter woesei* DSM 14684 genome (whose GC-content is 72%) and in the extremely low GC-content genome *Candidatus Carsonella* ruddii HT isolate Thao2000 genome (AT-rich with 85% AT in the genome; *Carsonella* genomes are known to be AT-rich [54]) are likely to belong to this case. However, the unknown false-CRISPRs remain to be further investigated.

## Conclusion

Using available complete archaeal and bacterial genomes, we systematically studied isolated CRISPRs (and *cas* loci) and false-CRISPRs. We demonstrated that it is important to differentiate isolated and false-CRISPRs, and our curation of false-CRISPRs could be used to reduce the false annotation of CRISPRs, useful for improving the annotation of CRISPR–Cas systems.

## Additional files

Additional file 1: A phylogenetic tree of 49*S. pyogenes* complete genomes. (DOCX 96 kb)
Additional file 2: An illustration of the CRISPR–Cas systems found in the *Z. mobilis* genomes. (DOCX 592 kb)
Additional file 3: A sequence file of real CRISPR arrays in the FASTA format. (TXT 9447 kb)
Additional file 4: The repeat sequences of false-CRISPRs in the FASTA format. (TXT 148 kb)
Additional file 5: A sequence file of false-CRISPRs in the FASTA format. Annotations of the false-CRISPRs are shown in the sequence headers. (TXT 2016 kb)
Additional file 6: False-CRISPR elements found in Biswas’ collection. (DOCX 33 kb)

## Abbreviations

CRISPR: Clustered regularly interspaced short palindromic repeats
false-CRISPR: Genomic elements that superficially reassemble CRISPRs but don’t contain diverse spacers and have no companion *cas* genes
STAR: *Staphylococcus aureus* repeat (STAR-like) element
