# Supplementary material for: Not all predicted CRISPR–Cas systems are equal: isolated cas genes and classes of CRISPR like elements
Source: BMC Bioinformatics. 2017 Feb 6;18:92. doi: 10.1186/s12859-017-1512-4 (PMC5294841; doi:10.1186/s12859-017-1512-4)
Supplement: Additional file 1: — A phylogenetic tree of 49S. pyogenes complete genomes. (DOCX 96 kb) [file 12859_2017_1512_MOESM1_ESM.docx]

**Additional file 1.** The phylogenetic tree of all 49 *Streptococcus pyogenes* complete genomes.
