# Supplementary material for: Not all predicted CRISPR–Cas systems are equal: isolated cas genes and classes of CRISPR like elements
Source: BMC Bioinformatics. 2017 Feb 6;18:92. doi: 10.1186/s12859-017-1512-4 (PMC5294841; doi:10.1186/s12859-017-1512-4)
Supplement: Additional file 2: — An illustration of the CRISPR–Cas systems found in the Z. mobilis genomes. (DOCX 592 kb) [file 12859_2017_1512_MOESM2_ESM.docx]

**Additional file 2**. CRISPR–Cas systems found in *Zymomonas mobilis* genomes. The phylogenetic tree of these genomes is shown on the left. The genomic context of CRISPR and *cas* genes is shown on the right. The symbol of “-//-” in the genomic context indicates that distance greater than 10,000bps. Genes are shown as arrows, including subtype I-F signature *cas* genes (shown in pink), subtype I-E signature *cas* genes (red), other *cas* genes (purple), and non-cas genes (gray). The blue blocks show the CRISPR loci and the numbers above the CRISPRS show the number of spacers.
