# Supplementary material for: Not all predicted CRISPR–Cas systems are equal: isolated cas genes and classes of CRISPR like elements
Source: BMC Bioinformatics. 2017 Feb 6;18:92. doi: 10.1186/s12859-017-1512-4 (PMC5294841; doi:10.1186/s12859-017-1512-4)
Supplement: Additional file 6: — False-CRISPR elements found in Biswas’ collection. (DOCX 33 kb) [file 12859_2017_1512_MOESM6_ESM.docx]

**Additional file 6.** The false-CRISPR elements found in Biswas’ collection (Biswas et al., 2014)

| **Genome ID** | **False-CRISPR region** | **Repeat sequence** | **Predicted Transcriptional direction (forward vs. backward)^*^** | **Supporting evidence forward strand^*^** | **Supporting evidence backward strand^*^** |
| --- | --- | --- | --- | --- | --- |
| NC_014957 | 47067-49232 | GTTCCGCACCCCTCCGGGGGTGCGGCACCATTGAAAC | F(HIGH) | motif(ATTGAAA.?) Secondary-Structure:[-16.00,-13.50] array-degeneracy:[0-3] | AT-flanking-region:[43.3-48.3] |
| NC_019387 | 58925-59293 | GTGTCCACCGTACCTTTAAGGGTTTGAAA | F(HIGH) | Secondary-Structure:[-2.50,-1.40] array-degeneracy:[0-10] AT-flanking-region:[55.0-41.7] | - |
| NC_009076 | 3948168-3948390 | GAGTCAGCGCTTCAGCGCT | R(HIGH) | longer-leader:[614,160] | AT-repeat:[3,4] array-degeneracy:[6-4] AT-flanking-region:[30.0-33.3] |
| NC_016771 | 1577783-1578123 | GTAGGTCCGGTGTTGCCAGTAGGTCC | R(MEDIUM) | array-degeneracy:[3-6] | AT-repeat:[3,7] Secondary-Structure:[-1.00,-3.70] AT-flanking-region:[36.7-56.7] |
| NC_014103 | 1377468-1377808 | GCGAGAAGAGCGGCGAGTAGAACGGCGAG | F(HIGH) | AT-repeat:[9,1] array-degeneracy:[0-6] | - |
| NC_008711 | 606879-607177 | CCACCCCCACGGTGACTGCAACT | R(LOW) | AT-repeat:[5,3] longer-leader:[1138,555] | array-degeneracy:[1-0] AT-flanking-region:[30.0-36.7] |
| NC_007434 | 182490-182713 | GAGTCAGCGCTTCAGCGCTT | R(MEDIUM) | longer-leader:[614,159] | AT-repeat:[3,5] array-degeneracy:[7-4] |
| NC_015163 | 46414-46666 | CGACGACATAGTTGAGCGATT | F(MEDIUM) | array-degeneracy:[0-4] longer-leader:[1355,122] | AT-flanking-region:[43.3-51.7] |
| NC_004368 | 1588776-1590517 | GTTGAGGCGCTAGTGCTGGCTGACGT | F(LOW) | Secondary-Structure:[-2.40,-1.40] array-degeneracy:[33-46] AT-flanking-region:[50.0-45.0] | AT-repeat:[3,7] longer-leader:[537,2370] |
| NC_013532 | 425538-425776 | AATAAGCTTAGAGCCATAGA | R(MEDIUM) | - | Secondary-Structure:[-0.30,-1.00] |
|  | 426586-426746 | TGAGCTTAGAGCCATAGACG | F(MEDIUM) | AT-repeat:[6,4] | - |
| NC_020126 | 5820445-5820709 | TCGCCCCTACCGCGCCGAACGTCTGCGTCGTGTTCGG | F(MEDIUM) | Secondary-Structure:[-5.80,-5.20] AT-flanking-region:[31.7-25.0] longer-leader:[1614,408] | AT-repeat:[3,8] |
| NC_014616 | 225803-228741 | GTGGTCAAAGTCATTGATCTGACAGGCATCTGAAAC | R(HIGH) | longer-leader:[341,29] | Secondary-Structure:[-2.70,-3.20] array-degeneracy:[1-0] AT-flanking-region:[45.0-56.7] |
| NC_016912 | 1734115-1734323 | ATAATGTGCAAGTTGGGGTAAGGGCCCCAACACAGA | F(MEDIUM) | AT-repeat:[12,6] Secondary-Structure:[-13.70,-11.30] array-degeneracy:[0-18] | AT-flanking-region:[50.0-78.3] |
|  | 2206999-2207192 | TGTTGGGGCCCCGCCAACTTGCA | R(LOW) | Secondary-Structure:[-2.90,-2.30] longer-leader:[357,143] | AT-repeat:[3,5] array-degeneracy:[8-1] |
| NC_007795 | 2335711-2335904 | TGTTGGGGCCCCGCCAACTTGCA | R(MEDIUM) | Secondary-Structure:[-2.90,-2.30] | AT-repeat:[3,5] array-degeneracy:[8-1] longer-leader:[61,143] |
|  | 1776486-1776694 | ATAATGTGCAAGTTGGGGTAAGGGCCCCAACACAGA | F(MEDIUM) | AT-repeat:[12,6] Secondary-Structure:[-13.70,-11.30] array-degeneracy:[0-18] | AT-flanking-region:[50.0-78.3] |
| NC_007760 | 4605448-4605577 | AATTACTCTGGGTCGGGGCCTC | R(MEDIUM) | - | AT-repeat:[3,6] |
| NC_013729 | 3468121-3468812 | CCGCAGCACCCGAGCCAGCCGCA | F(LOW) | AT-repeat:[5,] array-degeneracy:[10-13] longer-leader:[3530,1220] | Secondary-Structure:[-0.20,-1.50] AT-flanking-region:[23.3-30.0] |
| NC_018692 | 3959260-3959912 | TGCGCGCGCTTCTTCTTCCGCTT | R(LOW) | array-degeneracy:[0-7] longer-leader:[1033,126] | AT-repeat:[,9] AT-flanking-region:[50.0-63.3] |
| NC_012490 | 3337843-3338328 | GGAAGAGCGGTTGCCCTGGTATCC | R(HIGH) | - | AT-repeat:[4,5] Secondary-Structure:[-0.80,-1.50] AT-flanking-region:[26.7-31.7] longer-leader:[348,1616] |
|  | 2606281-2606538 | GCCAAGAAGGCTCCGGCCAAGACCGCAGCCAAG | R(LOW) | AT-repeat:[10,1] longer-leader:[546,209] | Secondary-Structure:[-5.10,-7.40] array-degeneracy:[1-0] |
| NC_019793 | 2503904-2504262 | GGTGGTTTCCGTCCCCGTGAAGACCG | R(MEDIUM) | array-degeneracy:[3-6] | AT-repeat:[3,6] Secondary-Structure:[-0.60,-2.30] AT-flanking-region:[33.3-38.3] |
| NC_020912 | 1704224-1705383 | TGCTGTTGCTGCTGGTCGAG | R(HIGH) | - | AT-repeat:[1,7] array-degeneracy:[7-5] AT-flanking-region:[43.3-48.3] longer-leader:[41,216] |
| NC_017860 | 194523-195602 | GTTGTTTTTACCTTGCAAACAGCAGGCAGAT | R(LOW) | Secondary-Structure:[-4.80,-4.30] | array-degeneracy:[3-0] AT-flanking-region:[55.0-65.0] |
| NC_017044 | 887821-887969 | GGAGGTGGAGGTGGTGATGG | R(HIGH) | - | AT-repeat:[3,4] array-degeneracy:[2-0] AT-flanking-region:[43.3-63.3] longer-leader:[498,1104] |
| NC_009089 | 3780590-3781770 | CCTGTTATTCCATTTGCTCC | R(HIGH) | - | array-degeneracy:[42-11] AT-flanking-region:[46.7-60.0] |
| NC_014550 | 3603165-3604002 | GTTCTGGAGTCGGCTCCACCGT | R(HIGH) | - | AT-repeat:[2,6] array-degeneracy:[13-1] AT-flanking-region:[36.7-48.3] longer-leader:[437,1710] |
|  | 2978505-2978821 | AGCGTTATCCGCAGTGTTCACATCCGTGT | R(HIGH) | - | AT-repeat:[5,9] longer-leader:[901,4622] |
| NC_014734 | 223908-225657 | GTTGTAGTTCCCTTCAATTTTGGGATAATCCACAAG | R(HIGH) | - | array-degeneracy:[12-3] AT-flanking-region:[61.7-68.3] |
| NC_018027 | 2336706-2337370 | GACGCCGCCTGCGCCGGGTGCGCCGGGTGTGCCGGTGG | F(LOW) | Secondary-Structure:[-12.00,-8.80] AT-flanking-region:[26.7-20.0] longer-leader:[2142,43] | AT-repeat:[1,5] array-degeneracy:[18-15] |
| NC_014025 | 51459-53316 | GGAGCTACCGGAATCACTGGAG | R(LOW) | AT-repeat:[6,3] | array-degeneracy:[50-11] AT-flanking-region:[38.3-48.3] |
| NC_013745 | 385695-385920 | TGTGCGATATGAATTAGCTG | F(HIGH) | array-degeneracy:[0-3] longer-leader:[328,85] | - |
| NC_009074 | 3872719-3872877 | GAGTCAGCGCTTCAGCGCT | F(LOW) | array-degeneracy:[0-4] AT-flanking-region:[46.7-33.3] | AT-repeat:[3,4] longer-leader:[20,160] |
| NC_015383 | 402907-403429 | CTCGATCTCCACGTGGAGAT | R(LOW) | array-degeneracy:[1-3] longer-leader:[686,159] | AT-repeat:[4,5] AT-flanking-region:[31.7-38.3] |
| NC_012468 | 1687993-1692511 | CTTGTGCTTGCTGAAGCCG | R(HIGH) | - | AT-repeat:[2,6] array-degeneracy:[142-113] longer-leader:[354,2177] |
| NC_018508 | 3223190-3224496 | ACTGGACCTCAAGGTCTTCAAGGTAACAC | R(HIGH) | - | Secondary-Structure:[-1.30,-4.80] array-degeneracy:[6-4] AT-flanking-region:[35.0-40.0] |
| NC_008086 | 946421-946668 | TTCAATCAAGGGACTTATAA | R(HIGH) | - | array-degeneracy:[4-0] longer-leader:[862,6278] |
| NC_017347 | 2387184-2387377 | TGTTGGGGCCCCGCCAACTTGCA | R(LOW) | Secondary-Structure:[-2.90,-2.30] longer-leader:[357,143] | AT-repeat:[3,5] array-degeneracy:[8-1] |
| NC_009975 | 214844-215069 | ATAATCAATTAAAAAGTGTGGTTATTCC | R(MEDIUM) | longer-leader:[812,293] | array-degeneracy:[8-0] |
| NC_019393 | 1641557-1641791 | TATAGAAGTACAGGTGTGGACAGACACTTAT | R(HIGH) | - | array-degeneracy:[9-1] AT-flanking-region:[56.7-68.3] |
| NC_016582 | 9669666-9669873 | GGGTGGTGTGGCCCAGCGG | R(HIGH) | - | AT-repeat:[1,3] array-degeneracy:[1-0] AT-flanking-region:[21.7-38.3] |
|  | 725593-726299 | CTTCCTTCTCCTTCTGTTCCTGCTCCTTCTCCTTC | R(HIGH) | - | AT-repeat:[,17] array-degeneracy:[39-33] longer-leader:[48,1403] |
| NC_017926 | 995729-995922 | AATTTCAATCAAGGGACTTATAA | R(HIGH) | - | motif(ATTGAAA.?) longer-leader:[854,6274] |
| NC_011658 | 1618961-1619301 | GTAGGTCCGGTGTTGCCAGTAGGTCC | R(MEDIUM) | array-degeneracy:[3-6] | AT-repeat:[3,7] Secondary-Structure:[-1.00,-3.70] AT-flanking-region:[36.7-56.7] |
| NC_018527 | 3961495-3961717 | GAGTCAGCGCTTCAGCGCT | R(HIGH) | longer-leader:[632,160] | AT-repeat:[3,4] array-degeneracy:[6-4] AT-flanking-region:[30.0-33.3] |
| NC_020892 | 675993-676425 | GTTTGAGAGTGTTGTCAAATACGAGGTAACTCAATC | F(LOW) | Secondary-Structure:[-2.70,0.00] array-degeneracy:[0-5] | AT-flanking-region:[48.3-68.3] longer-leader:[29,110] |
| NC_009484 | 1300540-1302395 | GCACCCGTCGCGCCGGTGGC | R(LOW) | array-degeneracy:[9-23] longer-leader:[1261,463] | AT-repeat:[1,2] AT-flanking-region:[36.7-48.3] |
| NC_002951 | 2325056-2325249 | TGTTGGGGCCCCGCCAACTTGCA | R(MEDIUM) | Secondary-Structure:[-2.90,-2.30] | AT-repeat:[3,5] array-degeneracy:[8-1] longer-leader:[61,143] |
| NC_015957 | 9970503-9970768 | CGGGAGCTCCGCCGGGAGCTGCG | F(HIGH) | Secondary-Structure:[-6.00,-4.40] array-degeneracy:[2-6] AT-flanking-region:[28.3-23.3] | longer-leader:[349,1276] |
| NC_017671 | 3586865-3587240 | CCTTCTTCACTGCCTTCTT | R(HIGH) | - | array-degeneracy:[7-2] AT-flanking-region:[30.0-40.0] longer-leader:[206,816] |
| NC_019439 | 157018-157216 | CATGGCTTCGCCACGCAGGCTATCAGAATTC | R(LOW) | AT-flanking-region:[70.0-61.7] longer-leader:[233,57] | Secondary-Structure:[-3.00,-4.40] array-degeneracy:[1-0] |
| NC_020990 | 3218016-3218434 | CCTCACGACGGTCACCACCACGG | F(LOW) | AT-repeat:[5,2] AT-flanking-region:[30.0-23.3] | array-degeneracy:[21-0] longer-leader:[697,1923] |
| NC_017200 | 1568487-1569448 | CCTTGAGGTCCTTGAGCACCAGTAGC | R(LOW) | array-degeneracy:[15-22] | AT-repeat:[5,6] Secondary-Structure:[-1.20,-2.20] |
| NC_008726 | 2281499-2281783 | GCGCCCGCCAAGAAGGCTCCGGCCAAG | R(LOW) | AT-repeat:[6,1] longer-leader:[542,106] | Secondary-Structure:[-3.00,-4.40] array-degeneracy:[4-0] AT-flanking-region:[28.3-35.0] |
| NC_015656 | 3841901-3842167 | CCCGCGCGCACGCGCGGCATGC | F(HIGH) | AT-repeat:[2,1] Secondary-Structure:[-3.30,-2.80] array-degeneracy:[1-5] AT-flanking-region:[35.0-20.0] | - |
| NC_015125 | 868066-868393 | GACCCGTCGCCGAAGTTCTGGAT | R(LOW) | array-degeneracy:[2-7] | AT-repeat:[4,5] AT-flanking-region:[21.7-28.3] |
| NC_014659 | 4376554-4376738 | CGCGCTTTGCGCACTTATCGC | R(MEDIUM) | AT-flanking-region:[28.3-18.3] | AT-repeat:[2,6] longer-leader:[70,1269] |
| NC_020520 | 2330345-2330505 | GGTGTGGTGCATGCCAACGG | R(LOW) | Secondary-Structure:[-1.00,0.00] longer-leader:[135,30] | AT-repeat:[3,4] array-degeneracy:[1-0] |
| NC_017030 | 8063924-8064269 | CGACGTCGCCACGGACTGGCAGGC | R(LOW) | AT-repeat:[4,2] | Secondary-Structure:[-1.30,-1.90] longer-leader:[1525,4114] |
|  | 8065555-8067184 | CGCAGCCCGAGTGGGCCAC | F(HIGH) | AT-repeat:[3,1] array-degeneracy:[23-31] AT-flanking-region:[28.3-21.7] longer-leader:[3156,1199] | - |
|  | 2120434-2121357 | GAAAACCTGTCGGACAGTCGGACAGGTTGGG | F(HIGH) | AT-repeat:[8,5] Secondary-Structure:[-10.80,-8.90] array-degeneracy:[9-30] AT-flanking-region:[25.0-21.7] | longer-leader:[49,113] |
|  | 2473025-2473676 | TGTCGGACAGTCGGACAGGTT | R(HIGH) | - | AT-repeat:[4,5] array-degeneracy:[3-0] |
|  | 859639-860354 | GACAGTCGGACAGGTTTTGGAGAACCG | F(MEDIUM) | AT-repeat:[7,5] AT-flanking-region:[35.0-28.3] longer-leader:[353,39] | array-degeneracy:[17-9] |
| NC_017831 | 4052411-4052633 | GAGTCAGCGCTTCAGCGCT | R(HIGH) | longer-leader:[614,160] | AT-repeat:[3,4] array-degeneracy:[6-4] AT-flanking-region:[30.0-33.3] |
| NC_017393 | 22568-22815 | AAAATAGATAATGTAGAAAA | R(HIGH) | - | array-degeneracy:[7-0] longer-leader:[149,1998] |
| NC_011247 | 94900-95098 | CACGATCATAATTTGGTCT | F(MEDIUM) | array-degeneracy:[0-5] | - |
| NC_003923 | 290103-290291 | TGCAAGTTGGCGGGGCCCCAACA | R(LOW) | AT-repeat:[5,3] AT-flanking-region:[68.3-55.0] | Secondary-Structure:[-2.30,-2.90] array-degeneracy:[10-0] longer-leader:[67,295] |
| NC_004193 | 3168886-3169178 | TTTGGGTTAGAGAGCTGCTCTATTGACTACTTT | R(LOW) | Secondary-Structure:[-4.30,-3.20] | array-degeneracy:[9-7] |
| NC_011725 | 4649631-4650255 | CAATTACTTCCACTTCTTC | F(HIGH) | array-degeneracy:[0-17] longer-leader:[2492,1182] | - |
| NC_014012 | 3459868-3460133 | AGGTTGAGATCTTTTTCCATCTCTTCATAGGA | R(HIGH) | - | Secondary-Structure:[-3.10,-5.10] array-degeneracy:[11-2] longer-leader:[345,773] |
| NC_007168 | 328283-336943 | AGCACATCATTGAGTGCGTCA | F(HIGH) | array-degeneracy:[130-153] longer-leader:[2135,877] | - |
| NC_003910 | 3967485-3968398 | ATATCATCTGGGTCTGTTAC | R(MEDIUM) | - | array-degeneracy:[6-1] |
| NC_015421 | 916-1070 | AACCCTTGCTACGAGCGAAAATCCGAAAATCGGAC | F(LOW) | Secondary-Structure:[-2.50,-0.70] | AT-flanking-region:[56.7-76.7] |
| NC_008607 | 189566-189823 | GTCTCCACTACAAAGCGGCGGAGCACGCATGTCAAC | R(LOW) | AT-repeat:[10,5] | AT-flanking-region:[46.7-56.7] longer-leader:[12,167] |
| NC_008312 | 2941350-2941874 | AGGTGTCAATTTTTTGAGGT | R(MEDIUM) | - | longer-leader:[77,2314] |
|  | 1606273-1607292 | TTTTTCAGTTATCAGCTTTT | F(MEDIUM) | array-degeneracy:[9-20] | longer-leader:[36,7876] |
| NC_018721 | 2613484-2614179 | GTTGTAACTGCCCTTATTTTGAAGGGTAAACACAGC | F(MEDIUM) | Secondary-Structure:[-8.40,-5.70] array-degeneracy:[0-5] | longer-leader:[145,314] |
|  | 2608898-2609328 | GTTGTAACTGCCCTTATTTTGAAGGGTAAACACAAC | F(HIGH) | Secondary-Structure:[-8.40,-5.70] array-degeneracy:[0-2] | - |
|  | 3964447-3964586 | TTGTGGCTGTGAGGCCCTGAAATAAATTCAGGGTGATAAG | R(LOW) | Secondary-Structure:[-11.50,-11.10] longer-leader:[1050,152] | array-degeneracy:[2-0] AT-flanking-region:[56.7-65.0] |
| NC_013929 | 5775873-5775997 | GGCGGGGCGGGCATGTGGCTGTC | R(HIGH) | - | AT-repeat:[1,4] longer-leader:[121,274] |
| NC_016935 | 6266467-6266714 | TAACACGGGTTTTCCGCGTTA | R(HIGH) | - | array-degeneracy:[8-0] AT-flanking-region:[35.0-40.0] |
|  | 3237103-3237315 | ATCTGTTGGCTAGTACAACC | F(LOW) | array-degeneracy:[0-3] | AT-flanking-region:[46.7-55.0] |
|  | 3288859-3289441 | AACACGGAAAATCCGTGTT | R(LOW) | array-degeneracy:[4-6] | AT-flanking-region:[31.7-40.0] longer-leader:[48,457] |
| NC_009648 | 1130143-1137307 | CCGACAGCGATTCGGATTCTGACAGCGA | F(MEDIUM) | AT-repeat:[7,5] array-degeneracy:[106-116] | longer-leader:[1111,4385] |
| NC_017017 | 5352-5506 | TCCGATTTTCGGATTTTCGCTCGTAGCAAGGGTTT | R(LOW) | AT-flanking-region:[76.7-56.7] | Secondary-Structure:[-0.70,-2.50] |
| NC_013062 | 869411-869673 | TTTTTGGCGCTCGCTTCGCTCGCGCCC | R(LOW) | AT-flanking-region:[60.0-51.7] longer-leader:[194,11] | AT-repeat:[,9] array-degeneracy:[2-1] |
| NC_013595 | 7296633-7296802 | GGACTGACCACGGGCGCCGGGCCG | F(HIGH) | AT-repeat:[3,1] Secondary-Structure:[-1.70,-1.10] array-degeneracy:[0-3] longer-leader:[354,61] | - |
|  | 6476434-6476582 | GGGCGGGACGGCACGCGGTC | F(LOW) | AT-repeat:[2,1] array-degeneracy:[0-2] | AT-flanking-region:[16.7-25.0] longer-leader:[165,671] |
|  | 10094996-10095189 | CCGTAGGCCTGCTGCTGGCCGTA | R(LOW) | array-degeneracy:[0-8] longer-leader:[1896,815] | AT-repeat:[2,5] AT-flanking-region:[21.7-30.0] |
|  | 9623281-9623508 | CACCGACGGCACCACCTGGGACTC | R(MEDIUM) | AT-repeat:[5,2] | Secondary-Structure:[-0.60,-1.20] array-degeneracy:[6-0] AT-flanking-region:[33.3-43.3] |
|  | 1047741-1050578 | GGTCCTGCCGGTGCGAGGGGTCCTGCGGGT | F(LOW) | Secondary-Structure:[-2.30,-0.50] array-degeneracy:[121-126] longer-leader:[715,92] | AT-repeat:[1,6] AT-flanking-region:[11.7-20.0] |
| NC_018693 | 2820385-2823755 | GGCGCTACTGGACCTACTGGACCT | F(MEDIUM) | array-degeneracy:[29-44] AT-flanking-region:[65.0-43.3] longer-leader:[586,192] | AT-repeat:[4,5] |
| NC_017367 | 987146-987394 | TTTCAATCAAGGGACTTATAA | R(HIGH) | - | motif(ATTGAAA.?) array-degeneracy:[2-0] AT-flanking-region:[55.0-61.7] longer-leader:[856,6273] |
|  | 119035-119287 | AGAACAAGAAAGGCAAAAAACA | F(LOW) | array-degeneracy:[3-5] | AT-flanking-region:[61.7-76.7] |
| NC_009641 | 2393437-2393630 | TGTTGGGGCCCCGCCAACTTGCA | R(LOW) | Secondary-Structure:[-2.90,-2.30] longer-leader:[357,143] | AT-repeat:[3,5] array-degeneracy:[8-1] |
|  | 1178380-1178566 | GGCGGGGCCCCAACATAGA | F(LOW) | AT-repeat:[5,1] AT-flanking-region:[70.0-58.3] | array-degeneracy:[3-1] |
| NC_014643 | 2368430-2369637 | CCCTCAATGAAAGTCACCCATTCTCATGGGTGAGAC | R(MEDIUM) | AT-repeat:[10,8] | Secondary-Structure:[-6.60,-8.00] array-degeneracy:[10-0] |
|  | 2360638-2360890 | CCCTCAATGAAAGTCACCTGTTCTCACAGGTGAGA | R(MEDIUM) | longer-leader:[132,2] | Secondary-Structure:[-5.60,-6.40] AT-flanking-region:[53.3-60.0] |
| NC_017093 | 6559915-6560212 | CTCGTCGTCCTCGTCGTCG | R(LOW) | array-degeneracy:[5-7] | AT-repeat:[,6] longer-leader:[58,612] |
| NC_015850 | 1236314-1236999 | GGTTTTCCCCCGCGCACGCGGGGACGAC | R(MEDIUM) | AT-flanking-region:[63.3-50.0] | AT-repeat:[3,4] array-degeneracy:[12-4] longer-leader:[46,107] |
|  | 1234131-1235254 | GGTTTTCCCCCGCGCACGCGGGGACGAC | R(HIGH) | - | AT-repeat:[3,4] array-degeneracy:[24-0] AT-flanking-region:[33.3-50.0] |
| NC_017033 | 2525823-2526172 | GCCTGGGACAAGACCAAGGA | R(LOW) | AT-repeat:[7,1] | array-degeneracy:[9-2] AT-flanking-region:[30.0-35.0] |
| NC_007633 | 339324-339586 | TTCAACCAACCCATTGGTAACTGAGACACCTCAAATGT | F(LOW) | array-degeneracy:[3-3] AT-flanking-region:[70.0-61.7] | Secondary-Structure:[-4.10,-4.90] |
| NC_019780 | 963104-963440 | AACCCAACAACAATCAGAAAGTAG | F(MEDIUM) | array-degeneracy:[5-6] | - |
|  | 2763290-2764455 | GACGATGACTATCTAGAAAA | F(MEDIUM) | array-degeneracy:[10-19] | - |
| NC_012914 | 546577-547162 | AATAAGACGGAAAAACCGTGCTACAG | R(LOW) | array-degeneracy:[4-16] | Secondary-Structure:[-3.60,-4.00] longer-leader:[35,336] |
|  | 5070518-5070744 | TAGCCGTAACCAGGTCCCATCTCTGCTGG | R(HIGH) | - | AT-repeat:[5,7] array-degeneracy:[3-0] AT-flanking-region:[36.7-41.7] |
| NC_010338 | 1704207-1704567 | GCCGGCTCGGGCGACGTCA | F(MEDIUM) | array-degeneracy:[4-7] longer-leader:[725,106] | AT-flanking-region:[38.3-46.7] |
| NC_015904 | 16783-16975 | TGTAGAAAAGAATTTAAAT | R(MEDIUM) | - | array-degeneracy:[2-0] |
| NC_008498 | 8727-8881 | TCCGATTTTCGGATTTTCGCTTGTAGCAAGGGTTT | R(LOW) | AT-flanking-region:[78.3-55.0] | Secondary-Structure:[-1.30,-2.50] |
| NC_019679 | 21556-22094 | CTTTACTAACCCCTAATCCCTAATAGGGATGGAAAC | R(LOW) | array-degeneracy:[0-2] longer-leader:[533,102] | Secondary-Structure:[-6.60,-8.80] AT-flanking-region:[48.3-58.3] |
| NC_008268 | 746300-746862 | CCGGCTCCTCCTCCTCGGGCTCCTCCTCC | R(MEDIUM) | array-degeneracy:[6-12] | AT-repeat:[,7] AT-flanking-region:[31.7-35.0] longer-leader:[477,1014] |
|  | 3918433-3918577 | GGCTGCGACGTGGTGGGCG | R(LOW) | array-degeneracy:[0-2] | AT-repeat:[1,3] AT-flanking-region:[15.0-23.3] |
| NC_010943 | 3719751-3720138 | CCTTCTTCACTGCCTTCTT | R(HIGH) | - | array-degeneracy:[7-1] AT-flanking-region:[30.0-41.7] |
| NC_017375 | 970769-970971 | TTTCAATCAAGGGACTTATAACTTTA | R(HIGH) | - | motif(ATTGAAA.?) array-degeneracy:[3-2] longer-leader:[859,6262] |
| NC_010531 | 206180-206352 | GCTGCAAAAAAGGTTGCTAAAAAGCG | R(MEDIUM) | - | array-degeneracy:[3-0] |
| NC_019683 | 814831-815131 | TCGCCACTTTAGTTAATCTCCAACA | F(LOW) | AT-flanking-region:[63.3-53.3] | longer-leader:[1027,2386] |
| NC_018503 | 5466-5650 | CCAGAAACTAAACCAGAGACTAAACCAGA | F(HIGH) | array-degeneracy:[0-2] AT-flanking-region:[66.7-60.0] | - |
| NC_015312 | 3052241-3053417 | TCACCAGCTCTTGTTGCGCAC | R(HIGH) | - | AT-repeat:[3,6] array-degeneracy:[11-6] AT-flanking-region:[23.3-36.7] longer-leader:[242,653] |
| NC_016001 | 2549401-2549896 | GTTGTAACTGCCCTTATTTTGAAGGGTAAACACAAC | F(HIGH) | Secondary-Structure:[-8.40,-5.70] array-degeneracy:[0-1] AT-flanking-region:[65.0-56.7] | - |
|  | 2550500-2550995 | GTTGTAACTGCCCTTATTTTGAAGGGTAAACACAAC | F(HIGH) | Secondary-Structure:[-8.40,-5.70] array-degeneracy:[1-2] AT-flanking-region:[66.7-56.7] | - |
| NC_016779 | 1382727-1383961 | CCAGTAGCGCCAGTAGCACC | F(HIGH) | AT-repeat:[5,2] array-degeneracy:[20-22] AT-flanking-region:[50.0-43.3] | - |
| NC_010079 | 2389073-2389266 | TGTTGGGGCCCCGCCAACTTGCA | R(MEDIUM) | Secondary-Structure:[-2.90,-2.30] | AT-repeat:[3,5] array-degeneracy:[8-1] longer-leader:[61,143] |
| NC_014389 | 336108-336644 | TTTTTTAAGGTTCTAAAAC | R(MEDIUM) | longer-leader:[351,26] | array-degeneracy:[4-3] |
| NC_016589 | 532740-533557 | GTTTCCTCCGCGTGGGCGGAGATAG | F(LOW) | Secondary-Structure:[-8.30,-7.20] array-degeneracy:[4-5] AT-flanking-region:[51.7-41.7] | AT-repeat:[3,6] longer-leader:[78,189] |

*: the information were listed in the Biswas’ collection (Biswas, et al., 2014) for CRISPR transcriptional direction. The fourth column shows the confidence level of the prediction in Biswas’ study. The fifth and sixth columns show the supporting evidence of each predicted strand.

**Reference**

Biswas, A. et al. (2014) Accurate computational prediction of the transcribed strand of CRISPR non-coding RNAs. *Bioinformatics*. 30, 1805-1813.
